# Severe Chronic Gingivitis in Association With Hypothyroidism and Grade 2 Adenoid Hypertrophy: A Case Report

**DOI:** 10.7759/cureus.49506

**Published:** 2023-11-27

**Authors:** Jyoti Khade, Ajay M Khade, Shefali Pantawane, Mangesh Phadnaik, Adiba Siddique, Gulshan R Bandre

**Affiliations:** 1 Periodontology, Government Dental College and Hospital, Nagpur, IND; 2 Pharmacology, Datta Meghe Medical College, Datta Meghe Institute of Higher Education and Research (Deemed to be University), Nagpur, IND; 3 Dentistry, Datta Meghe Medical College, Datta Meghe Institute of Higher Education and Research (Deemed to be University), Nagpur, IND; 4 Microbiology, Jawaharlal Nehru Medical College, Datta Meghe Institute of Higher Education and Research (Deemed to be University), Wardha, IND

**Keywords:** plaque, gingivitis, mouth breathing, adenoid hypertrophy, hypothyroidism

## Abstract

Hypothyroidism is the second-commonest endocrine disorder in the world. Similarly, gingivitis is also a highly prevalent oral condition in every population globally. Adenoid hypertrophy and associated mouth breathing may aggravate preexisting gingival inflammation. Here, we are presenting the case of a 22-year-old female gingivitis patient with bleeding from gums on the slightest provocation and with a two-year history of preexisting hypothyroidism. Thorough systemic examinations and investigations ruled out the presence of hematological and/or coagulation disorders. However, she was found to have grade 2 adenoid hypertrophy along with a habit of mouth breathing. Periodontal and systemic management of the patient has resolved her gingival bleeding to a greater extent. Still, there remain a lot of ambiguity and a lack of clarity about the exact etiology and mechanism of pathogenesis behind her oral and general health status. Cases like these pose a diagnostic challenge for the treating dentist or periodontist and thus require a coordinated and collaborative effort of multiple health specialties.

## Introduction

Gingivitis, a highly prevalent disease, is the non-specific inflammation of the gingiva, predominantly caused by local factors like dental plaque (biofilm) formation [1]. Gingivitis, for epidemiological study purposes, is assessed as ≥10% bleeding sites along with probing depths ≤3 mm [2]. It can be influenced and altered by systemic factors like pregnancy, puberty, menstrual cycle, and diabetes; medications like anticonvulsants, immunosuppressants, calcium channel blockers, and oral contraceptives; nutritional factors like vitamin C deficiency; and behavioral risk factors like smoking [3]. Gingival diseases can also be non-dental plaque-associated, like due to specific infections (bacterial, viral, or fungal origin), of genetic origin, and as a manifestation of systemic mucocutaneous lesions, allergic or foreign body reactions, and traumatic lesions [4]. In general, clinical features of gingivitis include changes in color and surface texture, edema, bleeding and discomfort on gentle probing, pain, halitosis, difficulty eating and brushing, and a reduced level of oral health-related quality of life. The presence of dental plaque and local plaque retentive factors is usually seen at the clinical sites of gingivitis [3].

In hypothyroidism, there is a decrease in thyroid hormone production and impaired thyroid gland function. It is a commonly occurring endocrinal disorder that affects primarily female adolescents and adults, with systemic manifestations like fatigue, weight gain, cold intolerance, joint and muscle pain, dry skin, and a reduced heart rate. The common oral findings in hypothyroidism include altered taste sensation, an enlarged tongue, periodontitis, altered tooth morphology, delayed wound healing, and a delayed rate of tooth eruption. The occurrence of severe chronic gingivitis in hypothyroidism is an uncommon observation. A reduction in tetraiodothyronine (T4) levels and raised thyroid-stimulating hormone (TSH) levels are indicative of hypothyroidism [5,6].

Adenoid hypertrophy is identified as a probable risk factor for dental caries, periodontal diseases, and halitosis. Mouth breathing has also been associated with chronic gingivitis, malocclusion, halitosis, and periodontitis. The simultaneous occurrence of adenotonsillar hypertrophy and mouth breathing may cause dry mouth, increased plaque accumulation, sleep disturbances, and dentofacial changes like class 2 division 1 malocclusion [7,8]. Surgical management like adenoidectomy, with or without tonsillectomy, usually leads to recovery from these complications and improvement in oral health [9,10]. Limited data is available in the literature about reports of simultaneous clinical presentations of hypothyroidism, adenoid hypertrophy, mouth breathing, and gingivitis. Here, we are reporting a case of severe generalized chronic gingivitis influenced by the presence of hypothyroidism, mouth breathing, and adenoid hypertrophy.

## Case presentation

A 22-year-old female patient reported to the department of periodontology with chief complaints of bleeding on the slightest provocation from gums and halitosis for the past six months. The patient first started noticing mild bleeding while brushing around two years ago, which had aggravated in the past six months. She had multiple visits to the local dental practitioners for similar complaints but got relief briefly after scaling. Due to spontaneous bleeding from gums, she was scared to brush her teeth, leading to inadequate cleaning as well as plaque and calculus deposition, further aggravating her periodontal condition. She also gave a history of herbal toothpaste usage for the last three years. Her decayed mandibular molar (tooth number 46) was treated endodontically four years ago. The patient was diagnosed with hypothyroidism two years ago and was taking a tablet of thyroxine sodium at 50 mg/day under the supervision of a general physician. This year (May 2023), the dose increased to 100 mcg, as her TSH levels were significantly higher.

**Figure 1:**
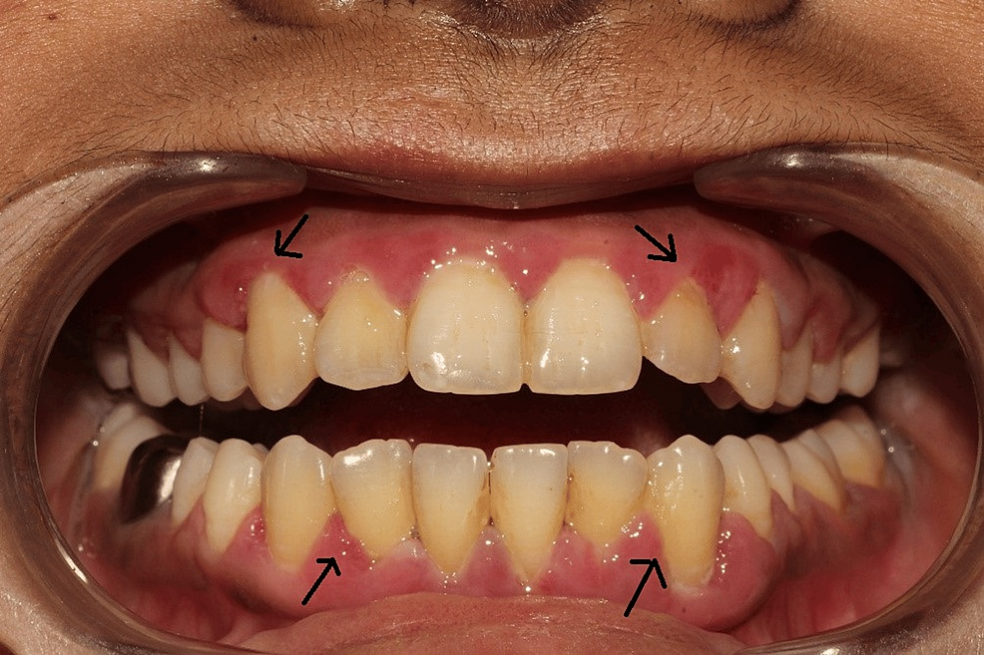
Intraoral clinical photograph at the first visit, showing all the classic signs of gingivitis

Extraoral examination revealed mild puffiness over the face and incompetent lips. Intraoral examination revealed generalized gingival inflammation (Figure 1). The gingiva was reddish pink in color, edematous, and soft in consistency. Spontaneous bleeding on probing with loss of stippling and exaggerated contours was noted, along with supra- and subgingival calculi, especially in the anterior region. Generalized pseudo pockets (≤3 mm) were seen on probing without clinical attachment loss (CAL) (except for 46 and 36, where CAL was 2 mm), which was primarily due to chronic inflammatory gingival enlargement. The patient had a mouth breathing habit that interfered with her sleep. Anterior open bite, edge-to-edge bite relation of the left side canines, and mild crowding in mandibular anterior teeth were also noted.

**Figure 2:**
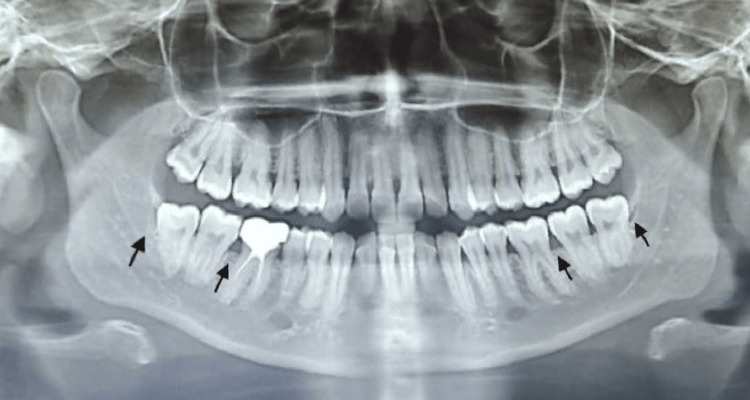
OPG revealed mild horizontal bone loss interproximally around 46 (which was endodontically treated four years back) and 36. Impacted mandibular third molars can also be seen OPG: orthopantomogram

**Figure 3:**
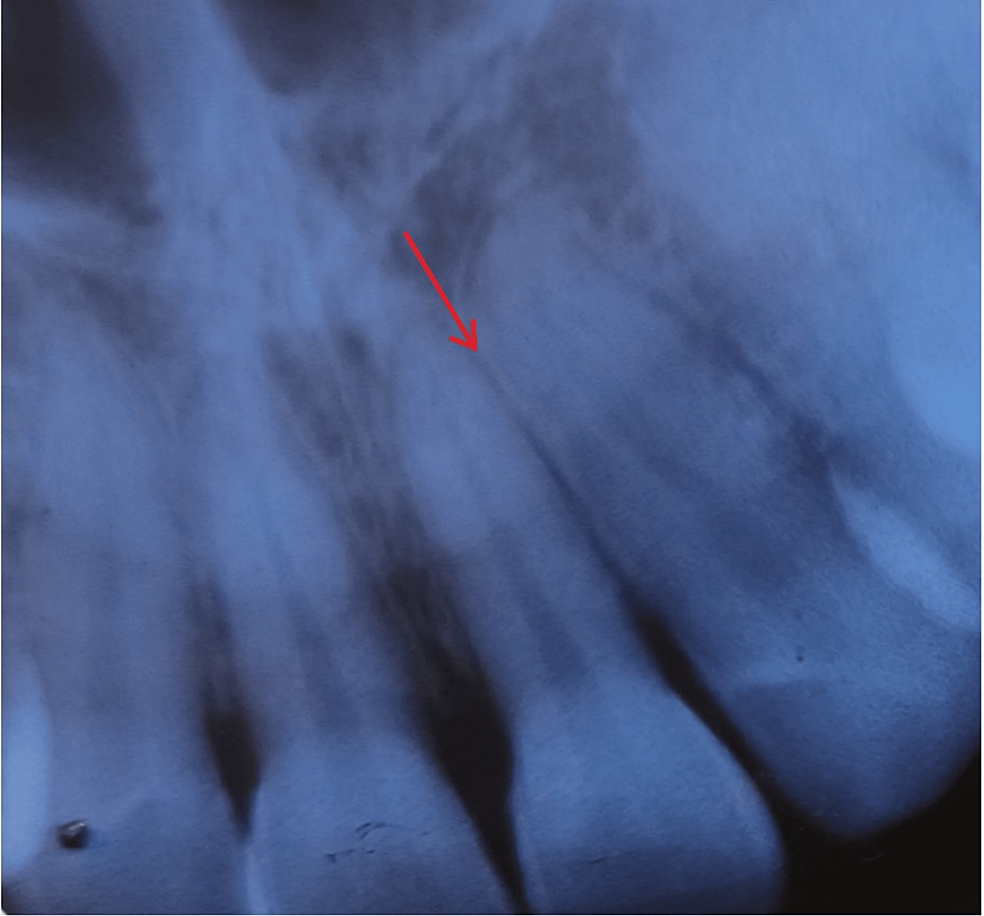
IOPA radiographs with maxillary anterior teeth (21 and 22) showing root proximity IOPA: intraoral periapical

Radiological examination through an orthopantomogram (OPG) revealed mild horizontal bone loss around the mandibular first molars and impaction with the mandibular third molars (Figure 2). Intraoral periapical (IOPA) radiographs of maxillary (Figure 3) and mandibular anterior teeth revealed root proximity.

Various investigations (Table 1) were advised to the patient, including a complete blood count, periodic thyroid function test, prothrombin time (PT), ultrasonography (USG) abdomen, liver function test (LFT), and blood glucose levels. While her USG report was regular, thyroid function tests (Table 2) revealed decreased triiodothyronine (T3) and elevated TSH levels. LFT reports and blood glucose levels were also within normal limits.

**Table 1:** Hematological investigations ALT: alanine transaminase; SGPT: serum glutamic pyruvic transaminase; AST: aspartate transaminase; SGOT: serum glutamic oxaloacetic transaminase; APTT: activated partial thromboplastin time; PT: prothrombin time; HCT: hematocrit

Investigations	Results	Normal range
Hemoglobin	11.3 gm/dl	12-15 gm/dl
Red blood cell count	4.31 million/cubic mm	3.8-4.8 million/cubic mm
HCT	35%	40-52%
Total leukocyte count	6700 cell/cubic mm	4000-10,000 cell/cubic mm
Platelet count	348,000/cubic mm	150,000-410,000/cubic mm
Bleeding time	4.30 minutes	2-9 minutes
Clotting time	7.04 minutes	4-10 minutes
Fasting blood glucose	85.64 mg/dl	70-100 mg/dl
Post-meal blood glucose	90.27 mg/dl	Up to 160 mg/dl
PT	16.9 seconds	14 seconds
APTT	50.7 seconds	34.0 seconds
Vitamin B12	308.0 pg/ml	211.0-911.0 pg/ml
Total bilirubin	0.6 mg/dl	0.3-1.2 mg/dl
Total proteins	8.9 mg/dl	6-8 mg/dl
Alkaline phosphates	131 U/L	70-306 U/L
ALT (SGPT)	17 U/L	˂45 U/L
AST (SGOT)	18 U/L	˂40 U/L

**Table 2:** Thyroid function test T3: triiodothyronine; T4: tetraiodothyronine; TSH: thyroid-stimulating hormone

Investigations	Results				Normal range
	November 2021	February 2022	March 2022	April 2023	
Serum T3	0.99	0.47	1.08	1.04	0.8-2.0 ng/ml
Serum T4	7.77	8.67	7.30	8.88	5.10-14.10 ug/dl
TSH	3.63	4.49	4.68	7.48	0.27-4.20 mIU/ml

Slightly raised PT and activated partial thromboplastin time (APTT) prompted us to get her thorough hematological investigations done to rule out any underlying coagulopathy or other hematological disorders. But all the results were within normal limits. Although an incisional biopsy of the gingiva is required to confirm the underlying cause and histopathology behind gingival bleeding, the patient and her parents were hesitant and unwilling to undergo the invasive minor surgical procedure.

Based on findings from history taking, intraoral examination, extraoral examination, and investigations, the patient was diagnosed as having severe generalized chronic gingivitis, modified by hypothyroidism, adenoid hypertrophy, and a lousy mouth breathing habit. The patient was initially informed and educated about the consequences of poor oral hygiene and the interrelationship of gingivitis with hypothyroidism, adenoid hypertrophy, and mouth breathing habits. The patient was primarily treated with non-surgical periodontal therapy, including oral hygiene instructions and full-mouth supragingival and subgingival scaling. Due to her highly inflamed intraoral status with bleeding on the slightest provocation, initially, she was prescribed topical metronidazole gel application twice daily for one week and chemical plaque control through chlorhexidine mouthwash 0.2% twice daily after meals for one week. An appointment was scheduled two days later for supragingival scaling. She was asked to refrain from using herbal toothpaste during periodontal treatment, as sometimes patients can be allergic to ingredients of the same type [11]. She was trained to use an ultra-soft toothbrush with the Charters toothbrushing technique.

One week after supragingival scaling, the patient was recalled for subgingival scaling and subgingival irrigation with chlorhexidine digluconate 0.2% solution. She was also prescribed vitamin C tablets and vitamin B complex supplements, prophylactically daily for one month, but it did not improve her oral conditions [12]. At the first-month follow-up, supra- and subgingival scaling was repeated, and oral hygiene instructions were reinforced. She was advised to visit the periodontology clinic every one to three months for recall evaluations and supportive periodontal treatment. But, sometimes, the patient needed help to attend the recall appointments due to her academic liabilities and financial constraints.

As the patient had a habit of mouth breathing, consultation from an ear, nose, and throat (ENT) specialist or otorhinolaryngologist was sought to rule out airway blockage before prescribing a habit-breaking appliance. During clinical examination and in X-ray nasopharynx lateral view, a mild deviated nasal septum (DNS) towards the right side was noticed. After the ENT check-up, an appointment for nasal endoscopy under local anesthesia was planned. The endoscopy revealed grade 2 adenoid hypertrophy, obstructing 50% of the nasal choanae and blocking the Eustachian tube opening. She has been advised steam inhalation, tablet montelukast 10 mg at bedtime, and nasal spray (consisting of corticosteroid fluticasone propionate) twice daily for one month, and the patient was kept on recall follow-up evaluations.

**Figure 4:**
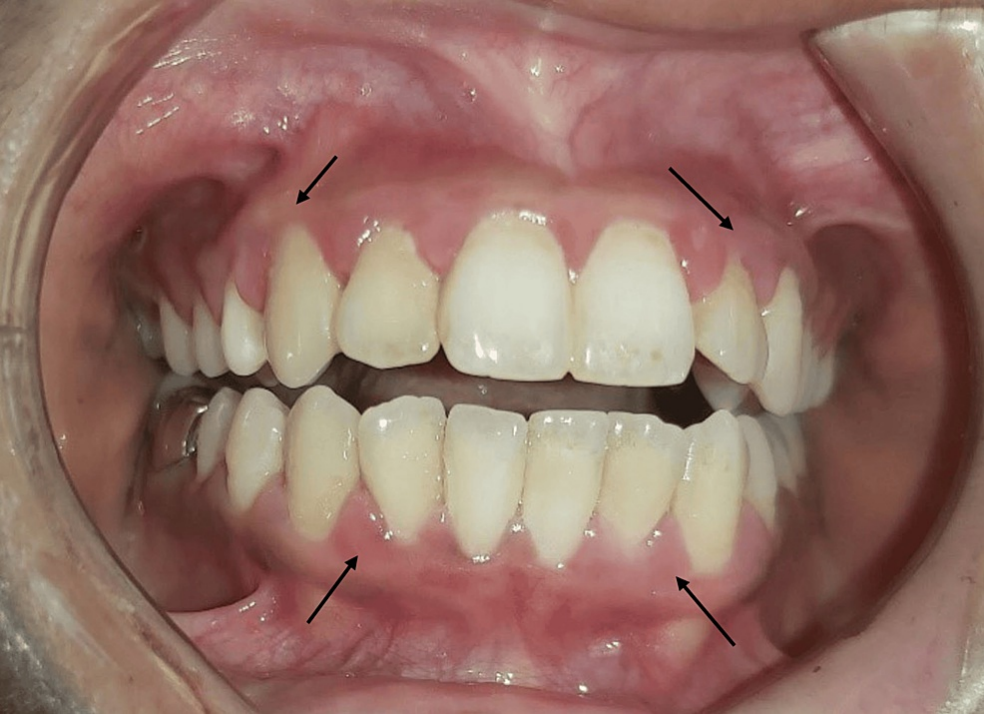
Immediately before supragingival scaling at one-and-half-year follow-up showing mild improvement in oral status

The ENT therapy has improved her symptoms of mouth breathing as well as her oral signs and symptoms of gingival bleeding. Hence, using steam inhalation, montelukast tablet, and nasal spray was advised to continue for one more month. The patient and her parents are satisfied with her condition's progress (Figure 4).

## Discussion

Gingival index and plaque index scores have been found to be significantly higher in children suffering from thyroid dysfunction in a study comparing the oral health status with healthy children [13]. Consistent with these findings, the present case also showed all signs of gingival inflammation with heavy plaque accumulation. 

A narrative review on the relationship between periodontal disease and Hashimoto's disease identified the possibility of an association between these two conditions, based on common etiopathological mechanisms including the proliferation of T-helper 1 and T-helper 17 lymphocytes, impaired vascular endothelial function in the microcirculation of the periodontium, and influence on bone metabolism [14]. Another scoping review also found a positive relationship between hypothyroidism and periodontitis [15]. The radiological examination of this patient also revealed localized mild horizontal bone loss in mandibular posterior teeth. Changes in the microcirculation of the periodontium can be the causative mechanism behind periodontal disease in thyroid dysfunction. Capillary alterations like reduced caliber and vascular modifications like increased number and tortuosity of capillary loops have been shown to be seen in the interdental papillae through gingival capillaroscopy in Hashimoto's thyroiditis patients as compared to healthy subjects [16].

Non-surgical periodontal therapy, consisting of oral hygiene instructions and full-mouth scaling and root planing, as shown in a clinical study, leads to improvement in the periodontal as well as thyroid status of patients suffering from hypothyroidism and periodontal disease simultaneously [17]. This patient is also being managed primarily by conservative non-surgical periodontal therapy at one- to three-month intervals because of her increased tendency of bleeding and heavy plaque deposition.

Sometimes, von Willebrand factor and factor VIII procoagulant activity (VIII:C) are reduced, and bleeding time may be elevated in hypothyroidism patients [18]. Preexisting hypothyroidism can affect oral health status as evident in literature. A delay in diagnosing and treating hypothyroidism may lead to decrease in activity as well as serum levels of von Willebrand factor causing prolonged bleeding, as reported in a case of dental extraction [19]. But this patient had all her hematological and coagulation markers within normal limits.

During her recall evaluation, this patient was diagnosed with grade 2 adenoid hypertrophy, leading to blockage of airway and obstructive sleep apnea (OSA). This can be the reason behind her mouth breathing habit as well as increased severity of gingival inflammatory signs in maxillary anterior teeth as compared to mandibular anterior teeth. An observational study has recently shown prevalence of hypothyroidism in patients simultaneously suffering from periodontitis and OSA [20].

An animal study has revealed that changes in the levels of thyroid hormones may influence periodontitis by enhancing alveolar bone loss through an increase in the number of resorbing cells [21]. This information is important from prognosis point of view for the current patient as she is a case of a severe type of generalized chronic gingivitis with heavy plaque formation, oral dryness due to mouth breathing, adenoid hypertrophy, and hypothyroidism. Her oral and systemic health makes her susceptible for the development of periodontitis, thereby highlighting the need for a customized aggressive type of periodontal therapy measures.

## Conclusions

The present case report highlights the diagnostic implications and challenges faced in systemic conditions like hypothyroidism and adenoid hypertrophy simultaneously occurring in periodontal disease patients. Management of the patient for oral symptoms and systemic conditions requires a collaborated effort on the part of the dentist/periodontist, general physician, ENT specialist, nutritionist, fitness expert, social worker, and behavioral counsellors. Hence, holistic approach in such cases becomes crucial to satisfactorily manage and maintain a controlled oral and systemic status.
